# Astragalus-Containing Chinese Herbal Medicine Combined With Chemotherapy for Cervical Cancer: A Systematic Review and Meta-Analysis

**DOI:** 10.3389/fphar.2021.587021

**Published:** 2021-07-30

**Authors:** Lei Shen, Si Ra Gwak, Zhen Yang Cui, Jong Cheon Joo, Soo Jung Park

**Affiliations:** ^1^ Aerospace Center Hospital, Beijing, China; ^2^ Department of Sasang Constitutional Medicine, College of Korean Medicine, Wonkwang University, Iksan, South Korea; ^3^ Rehabilitation Medicine College, Weifang Medical University, Weifang, China; ^4^ Department of Sasang Constitutional Medicine, College of Korean Medicine, Woosuk University, Jeonju, South Korea

**Keywords:** astragalus, chemotherapy, Chinese herbal medicine, cervical cancer, systematic review, meta-analysis

## Abstract

**Background:** Cervical cancer is the fourth most common malignant tumor among women worldwide. This study aimed to evaluate the efficacy of Astragalus-containing Chinese herbal medicine (CHM) combined with chemotherapy (CT) for the treatment of cervical cancer.

**Methods:** Ten electronic databases including PubMed, Cochrane Library, Embase, Korean databases, and Chinese medical databases, were systematically searched up to July 2020. All randomized controlled trials using Astragalus-containing CHM combined with CT to treat cervical cancer were included.

**Results:** A total of 19 trials were included in the analysis. Compared with the control group, the Astragalus-containing CHM combined with CT group showed a significantly increased tumor response (complete and partial response (CR and PR)) (risk ratio [RR] = 1.25, 95% confidence interval [CI]: 1.17–1.33, *p <* 0.00001) and Karnofsky performance score (KPS) (standardized mean difference [SMD] = 1.81, 95% CI: 1.46–2.17, *p <* 0.00001). This group also displayed remarkably reduced CT toxicity.

**Conclusion:** Our study suggests that Astragalus-containing CHM might be a potential option for cervical cancer to enhance the curative efficacy and reduce CT toxicity.

## Introduction

Cervical cancer is the fourth most common malignant tumor among women worldwide, with almost 0.6 million cases and 0.3 million deaths per year (Arbyn et al., 2020). Despite advances in early screening methods, the incidence of cervical cancer is still high, particularly in some low- and middle-income countries (Torre et al., 2016). Despite considerable advances in the treatment of cervical cancer, its clinical application is limited by surgery-related complications, disease recurrence, and therapy-related side effects. It is well-known that chemotherapy (CT) is usually accompanied by adverse events including hematologic and gastrointestinal toxicity (Chemoradiotherapy for Cervical Cancer Meta-Analysis, 2008). Conventional symptomatic therapy to reduce side effects is commonly used, but its effect is not significant.

Due to its efficacy and low toxicity, Chinese herbal medicine (CHM) has been widely used in cancer treatment for many years. Of particular interest is the herb Astragalus, which has antitumor, antioxidant stress, hepatoprotection, neuron protection, and other pharmacological effects (Li et al., 2014). In antitumor studies, it has been reported that Astragalus can enhance cellular and humoral immune functions, suppress the growth of tumors, and reduce CT-induced injury (Liu et al., 2017; Hui et al., 2020). Lin et al. reviewed the efficacy and safety of Astragalus-containing CHM combined with CT for patients with colorectal cancer and their results show that Astragalus-containing CHM combined with CT is more effective than CT alone (Lin et al., 2019). In addition, numerous studies have reported the effectiveness of Astragalus-containing CHM as an adjuvant therapy for cancer (Xiangyong et al., 2016). However, there have been no systematic reviews or meta-analyses on the efficacy of Astragalus-containing CHM in patients with cervical cancer.

This study aimed to investigate the effect of Astragalus-containing CHM combined with CT on tumor response, quality of life, and reduction of side effects in patients with cervical cancer.

## Material and Methods

### Data Sources and Search Strategy

The search was conducted in the following ten electronic databases to July 2020: PubMed, Cochrane Library, Embase, China National Knowledge Infrastructure (CNKI), Wanfang, Journal Integration Platform (VIP), KMbase, National Discovery for Science Leaders (NDSL), Oriental Medicine Advanced Searching Integrated System (OASIS), and Korean Studies Information Service System (KISS). In addition, we searched the references of all included studies by hand, grey literature, dissertations, letters, government documents, research reports, conference proceedings, and abstracts to avoid publication bias. “Uterine cervical neoplasms” and “Astragalus or Chinese herbal medicine” were the main keywords in our search strategy.

### Eligibility Criteria

#### Types of Studies

Only two-arm randomized controlled trials (RCTs) were eligible. Non-RCTs, quasi-RCTs, *in vitro*, and animal studies were excluded. Duplicated publications, case reports, reviews, and abstracts were also excluded.

#### Types of Participants

Eligible studies included women with a clear diagnosis of cervical cancer confirmed by pathological sections. In addition, all participants in the treatment and control groups were treated with CT. No restrictions were placed on age, ethnicity, degree of pain, or disease duration.

#### Types of Interventions

Patients in the treatment group were treated with Astragalus-containing CHM combined with CT, while the control group was treated with CT only. Included studies used Astragalus-containing CHM in various forms, such as decoctions, capsules, and tablets. Studies using intravenous administration were excluded. Both monochemotherapy and polychemotherapy were included, as well as combination therapy with radiotherapy; Chinese nonherbal medicinal therapies such as acupuncture, cupping, or point application were excluded.

#### Types of Outcome Measures

Tumor response and Karnofsky performance score (KPS) were the primary outcomes. The secondary outcome was a reduction in CT toxicity.

Based on the WHO scale, the tumor response to Astragalus-containing CHM in cervical cancer patients with complete response (CR), partial response (PR), stable disease (SD), and progressive disease (PD) was investigated. The improved or stable performance status of cervical cancer patients was examined according to the KPS, in which 100 refers to a normal patient without any complaints, 70 refers to a patient unable to carry on normal activity, 50 refers to a patient who requires considerable assistance, 40 refers to a disabled patient, and 30 refers to a hospitalization-recommended patient (Zhu et al., 2016). CT toxicity includes nausea and vomiting, hair loss, neurotoxicity, and hepatic and renal toxicities.

### Study Selection and Data Extraction

Two reviewers independently screened the articles according to the inclusion and exclusion criteria and extracted data based on a standardized data collection form. Disagreements between the two reviewers were resolved by either consensus or the inclusion of a third reviewer. The following data were extracted: first author, year, sample size, patient characteristics, intervention details, and outcomes.

### Quality Assessment

Two independent reviewers assessed the methodological quality using the Cochrane risk of bias (RoB) tool (Higgins, 2008). Disagreements between the two reviewers were resolved by discussion with a third reviewer. The following items were used to assess the methodological quality of RCTs: random sequence generation, allocation concealment, blinding of participants and personnel, blinding of outcome assessment, incomplete outcome data, selective reporting, and other biases.

### Statistical Analysis

RevMan 5.3 software of the Cochrane Collaboration was used for data analysis. For dichotomous data, a risk ratio (RR) with a 95% confidence interval (CI) was reported. For continuous variables, a standardized mean difference (SMD) with a 95% CI was reported. We used a fixed-effects model to estimate treatment effects. Heterogeneity was assessed using the *I* ^*2*^ statistic and *I* ^*2*^ > 50% was assumed to have high heterogeneity. A *p*-value < 0.05 was considered statistically significant. A funnel plot was used to analyze publication bias among the included studies.

## Results

**FIGURE 1:**
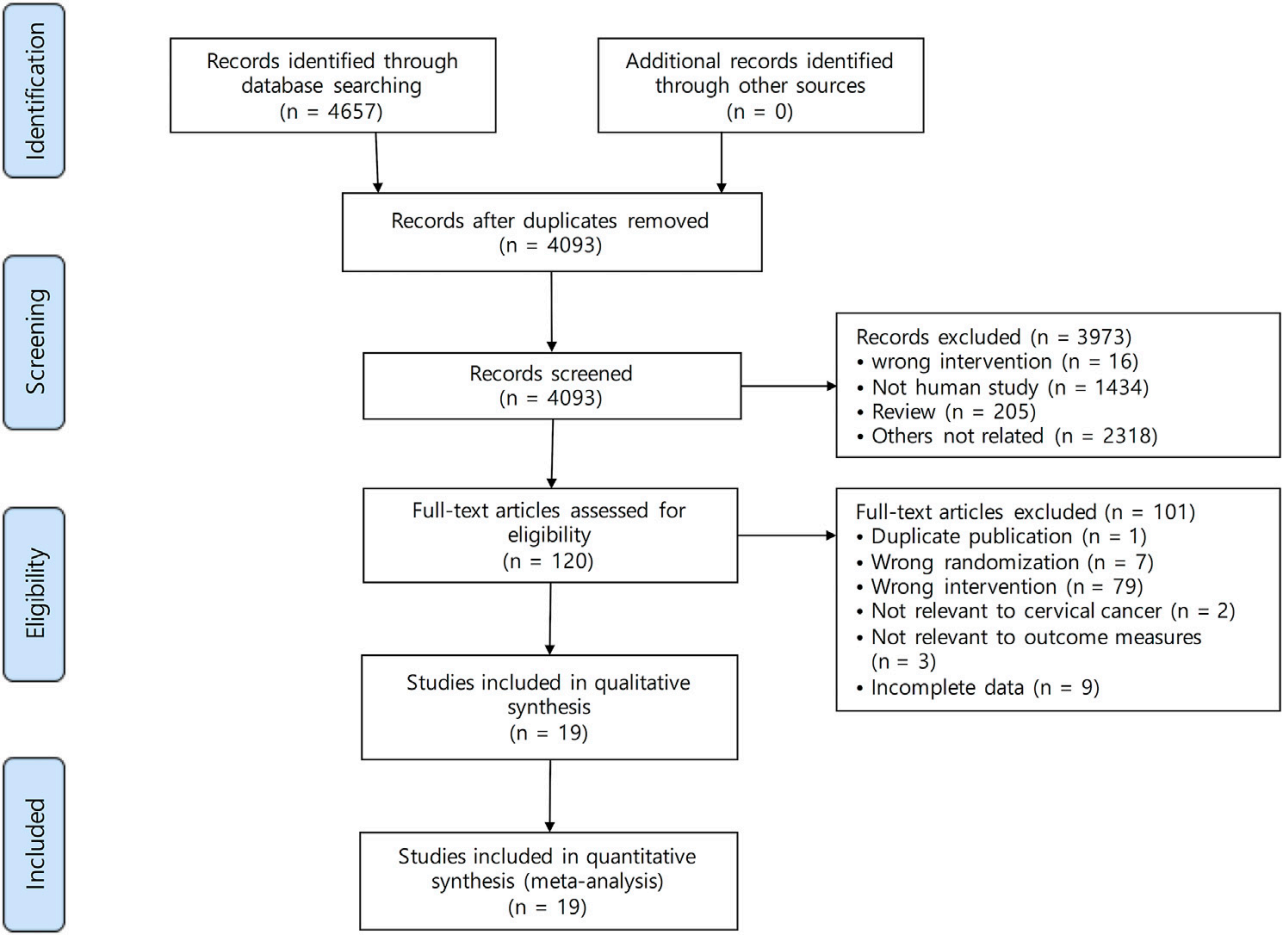
Study flow diagram.

A total of 4,657 studies were identified by searching PubMed (*n* = 176), the Cochrane library (*n* = 181), Embase (*n* = 2,260), CNKI (*n* = 390), the Wanfang database (*n* = 1,483), VIP (*n* = 6), KISS (*n* = 53), KMbase (*n* = 27), NDSL (*n* = 81), and OASIS (*n* = 0). Figure 1 shows the screening process. A total of 564 articles were excluded following screening for duplicates. After reviewing the titles and abstracts, 3,973 studies were excluded because they did not meet the criteria. The full texts of 120 studies were reviewed, and 19 studies (Wang, 2014; Jian et al., 2015; Li and Duan, 2015; Li and Su, 2015; Yang, 2015; Qin et al., 2016; Wu, 2017; Yang, 2017; Chang et al., 2018; Xu and Qi, 2018; Yang and Wang, 2018; Guan et al., 2019; Liu, 2019; Sun et al., 2019; Wen and Ding, 2019; Xu et al., 2019; Zhu and Li, 2019; Zuo, 2019; Zhang, 2020) were included in this systematic review and meta-analysis.

### Study Characteristics

All included studies were conducted in China and published in Chinese between 2014 and 2020. A total of 19 studies with 1,649 patients were analyzed; 827 patients were in the Astragalus-containing CHM combined with CT group, and the other 822 patients were in the CT alone group. Table 1 shows the characteristics of the studies, including sample size, age, duration, and outcomes. Astragalus-containing CHM used in the included studies were Fuzheng Guben decoction, Fuzheng Peiben decoction, modified Renshen Yangrong decoction, and so on (Supplementary Table 1).

**TABLE 1:** Characteristics of included studies.

Study	Sample size (TG/CG)	Age (years)	TNM stage (no. of patients)	Intervention group	Control group	Outcomes	Duration
Chang et al. (2018)	84 (42/42)	TG: 48.75 ± 10.24	TG: III, 26; IV, 16	Astragalus-containing CHM + TP	TP	Tumor response	4 weeks * 3 courses
		CG: 48.25 ± 10.13	CG: III, 25; IV, 17				
Guan et al. (2019)	92 (46/46)	TG: 35.74 ± 3.62	Stage was more than II A	Astragalus-containing CHM + Pac + CPT	Pac + CPT	Tumor response	3 weeks * 2 courses
		CG: 32.18 ± 2.76					
Jian et al. (2015)	41 (21/20)	TG: 52.38 ± 8.09	TG: IIIA, 2; IIIB 3; IVA, 3; IVB, 13	Astragalus-containing CHM + DP	DP	1. Tumor response	CT: 21d * 2 courses Astragalus-containing CHM: 8 weeks
		CG: 50.55 ± 9.81	CG: IIIA, 3; IIIB 4; IVA, 2; IVB, 11			2. KPS	
						3. Chemotoxicity	
Li and Duan (2015)	87 (45/42)	56.5 ± 10.6	IB, 46; IIA, 41	Astragalus-containing CHM + TC	TC	Chemotoxicity	3 weeks * 1 courses
Li and Su (2015)	90 (45/45)	TG: 55.6 ± 8.7	TG: IB, 9; IIA, 8; IIB, 24; III, 4	Astragalus-containing CHM + TC	TC	Chemotoxicity	3 weeks * 1 courses
		CG: 54.8 ± 8.4	CG: IB, 8; IIA, 9; IIB, 21; III, 7				
Liu (2019)	100 (50/50)	TG: 54.11 ± 2.42	TG: IIB, 21; III, 19; IV, 10	Astragalus-containing CHM+ 5-FU + CPA + pingyangmycin	5-FU + CPA + pingyangmycin	Tumor response	8 weeks
		CG: 54.25 ± 2.47	CG: IIB, 20; III, 20; IV, 10				
Qin et al. (2016)	60 (30/30)	TG: 48.34 ± 5.35	TG: IIB, 18; IIIA, 5; IIIB, 7	Astragalus-containing CHM + Pac + CPT	Pac + CPT	1. Tumor response	Not reported
		CG: 50.45 ± 5.85	CG: IIB, 16; IIIA, 6; IIIB, 8			2. KPS	
						3. Chemotoxicity	
Sun et al. (2019)	116 (58/58)	TG: 55.46 ± 13.27	TG: IIB, 32; IIIA, 14; IIIB, 12	Astragalus-containing CHM + TO	TO	1. Tumor response	CT: 21 days * 1∼2 courses
		CG: 55.23 ± 13.12	CG: IIB, 33; IIIA, 12; IIIB, 13			2. KPS	Astragalus-containing CHM: 10 days * 2 courses
						3. Chemotoxicity	
Wang (2014)	90 (45/45)	TG: 53.2 ± 7.5	TG: IIB, 25; IIIA, 9; IIIB, 11	Astragalus-containing CHM + Pac + CPT	Pac + CPT	1. Tumor response	Unclear
		CG: 53.5 ± 7.8	CG: IIB, 27; IIIA, 8; IIIB, 10			2. KPS	
						3. Chemotoxicity	
Wen and Ding (2019)	114 (57/57)	TG: 56.37 ± 7.93	TG: IIA, 19; IIB, 20; IIIA,18	Astragalus-containing CHM + TP	TP	1. Tumor response	7 days * 3 courses
		CG: 55.49 ± 7.62	CG: IIA, 19; IIB, 21; IIIA, 17			2. Chemotoxicity	
Wu (2017)	80 (40/40)	Total: 56.3 ± 7.2	IIB 26, IIIA, 38, IIIB, 16	Astragalus-containing CHM + Pac + CPT	Pac + CPT	1. Tumor response	Unclear
						2. Chemotoxicity	
Xu and Qi (2018)	90 (45/45)	TG: 53.3 ± 7.6	TG: IIB, 23; IIIA, 10; IIIB, 12	Astragalus-containing CHM + Pac + CPT	Pac + CPT	1. Tumor response	Unclear
		CG: 53.1 ± 7.4	CG: IIB, 24; IIIA, 10; IIIB, 11			2. Chemotoxicity	
Xu et al. (2019)	60 (30/30)	TG: 50.13 ± 14.26	TG: IB1, 13; IB2, 10; IIA, 5; IIB, 2	Astragalus-containing CHM + Pac + CPT	Pac + CPT	Tumor response	Unclear
		CG: 50.27 ± 13.49	CG: IB1, 14; IB2, 10; IIA, 4; IIB, 2				
Yang (2015)	80 (40/40)	TG: 58.7 ± 7.9	TG: IB, 7; IIA, 5; IIB, 19; IIIA, 5; IIIB, 4	Astragalus-containing CHM + MCF	MCF	1. Tumor response	CT: 3 weeks * 3 courses
		CG:59.4 ± 6.1	CG: I B, 6; IIA, 4; IIB, 20; IIIA, 6; IIIB, 4			2. Chemotoxicity	Astragalus-containing CHM: 9 weeks
Yang (2017)	119 (60/59)	TG: 55.37 ± 12.82	TG: IIA, 18; IIB, 30; III 12	Astragalus-containing CHM + TO	TO	Tumor response	3 months
		CG: 54.86 ± 12.97	CG: IIA, 17; IIB, 28; III 14				
Yang and Wang (2018)	62 (31/31)	TG: 48.24 ± 1.13	TG: IIB, 12; IIIA, 5; IIIB, 14	Astragalus-containing CHM + TP	TP	1. Tumor response	CT: 5 days
		CG: 48.64 ± 1.33	CG: IIB, 10; IIIA, 3; IIIB, 18			2. Chemotoxicity	Astragalus-containing CHM: 10 days
Zhu and Li ( 2019)	92 (46/46)	TG: 54.03 ± 7.12	TG: IIB, 31; III, 15	Astragalus-containing CHM + DP	DP	1. Tumor response	4 weeks * 3 courses
		CG: 53.29 ± 6.37	CG: IIB, 32; III, 14			2. Chemotoxicity	
Zuo (2019)	86 (43/43)	TG: 46.53 ± 3.27	TG: IIB, 18; IIIA, 13; IIIB, 12	Astragalus-containing CHM + Pac + CPT	Paclitaxel + CPT	1. Tumor response	21 days * 2 courses
		CG: 46.62 ± 3.87	CG: IIB, 17; IIIA, 15; IIIB, 11			2. Chemotoxicity	
Zhang (2020)	106 (53/53)	46.77 ± 4.52	IIIA, 68; IIIB, 38	Astragalus-containing CHM + TP	TP	Tumor response	4 weeks * 3 courses

CG, control group; CHM, Chinese herbal medicine; CPA, cyclophosphamide; CPT, irinotecan; CT, chemotherapy; DP, cisplatin + docetaxel; KPS, Karnofsky performance scale; MCF, mitomycin + cisplatin + 5-fluorouracil; Pac, paclitaxel; TC, paclitaxel + carboplatin; TG, treatment group; TO, paclitaxel + oxaliplatin; TP, cisplatin + paclitaxel; 5-Fu, 5-fluorouracil.

### Risk of Bias in Included Studies

**FIGURE 2:**
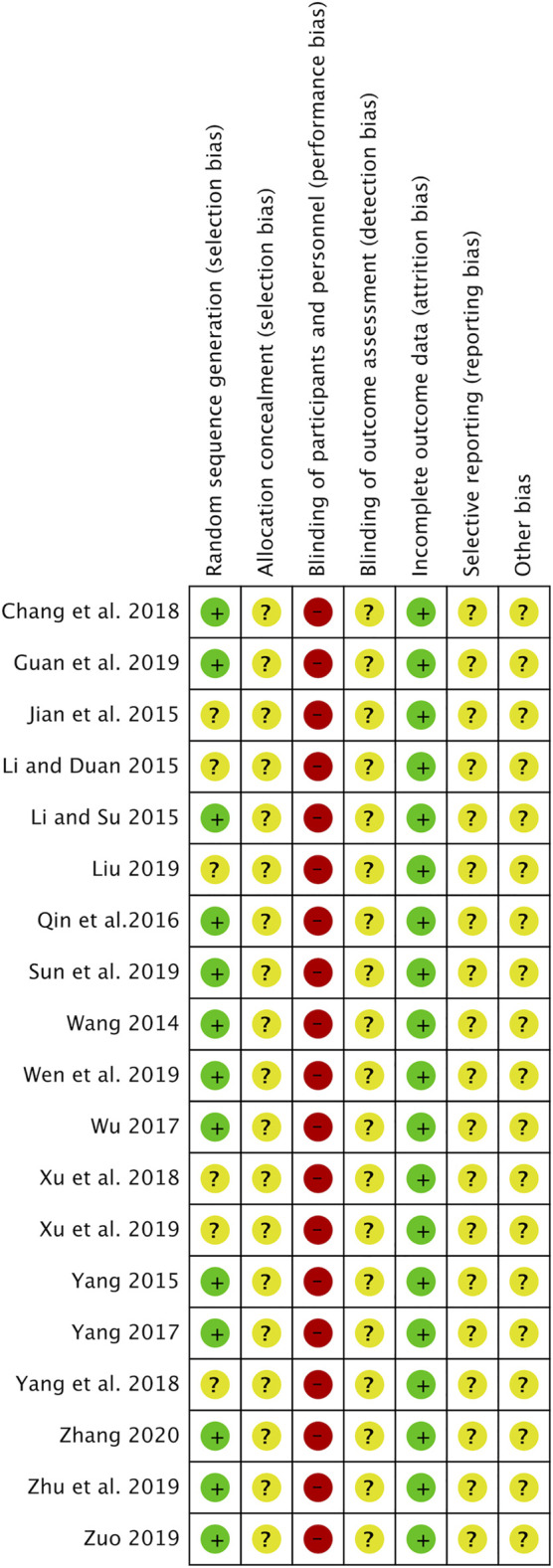
Risk of bias (RoB) summary.

Figure 2 shows the RoB in the included studies. All studies were described as randomized, and 13 studies (Wang, 2014; Li and Su, 2015; Yang, 2015; Qin et al., 2016; Wu, 2017; Yang, 2017; Chang et al., 2018; Guan et al., 2019; Sun et al., 2019; Wen and Ding, 2019; Zhu and Li, 2019; Zuo, 2019; Zhang, 2020) used random number tables. None of the studies used allocation concealment. All studies had a high RoB in the blinding of participants and personnel because they did not use a placebo in the control group. All studies were unclear on the blinding of outcome assessment and the selective reporting outcome. Other biases were also evaluated as unclear in all studies due to insufficient information.

### Meta-Analysis

#### Tumor Response

**FIGURE 3:**
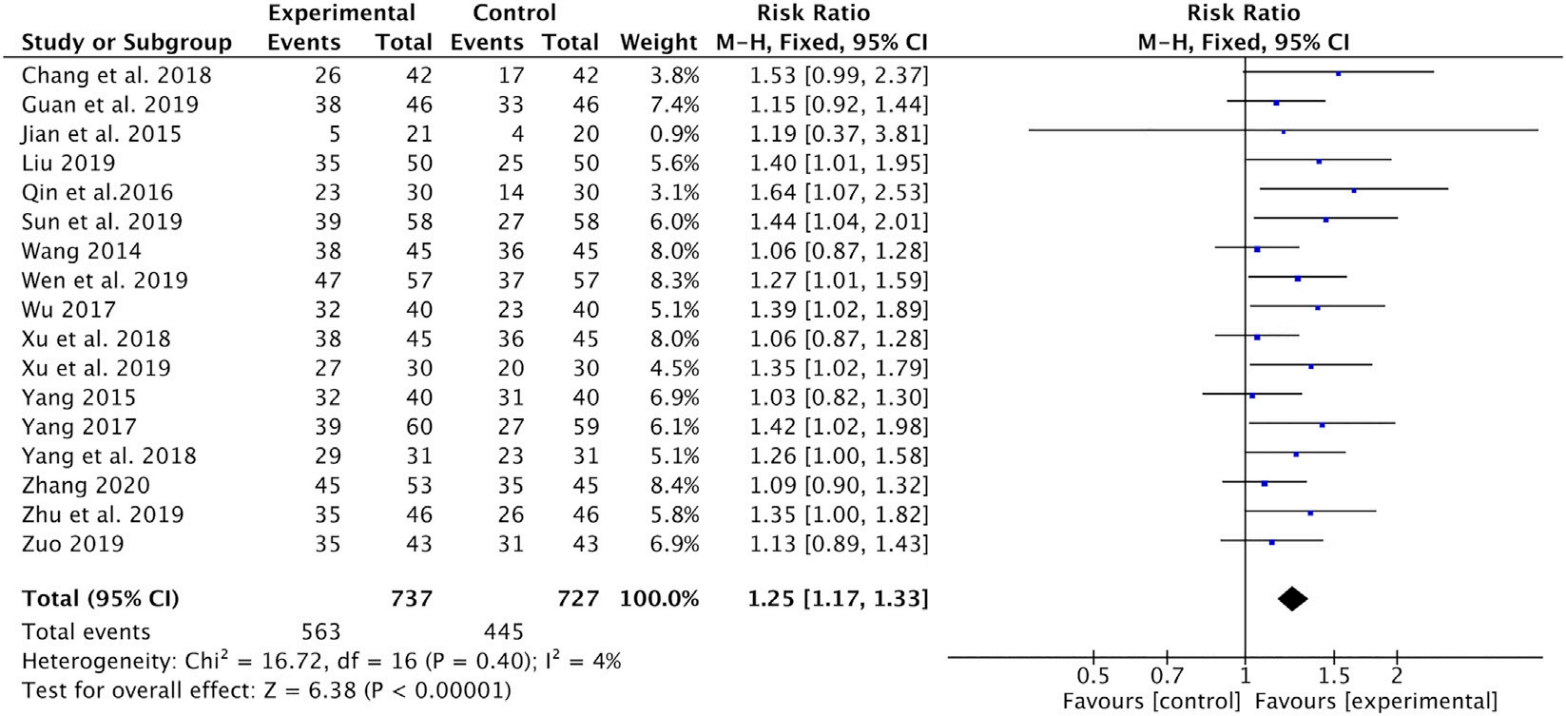
Forest plot of tumor response (CR + PR) of Astragalus-containing CHM combined with CT vs. CT alone. CHM, Chinese herbal medicine; CT, chemotherapy; CR, complete response; PR, partial response.

As shown in Figure 3, Astragalus-containing CHM therapy was associated with a significant increase in the number of patients who reported complete or partial response (CR and PR) (RR = 1.25, 95% CI: 1.17–1.33, *p <* 0.00001, *I* ^*2*^ = 4%) (Wang, 2014; Jian et al., 2015; Yang, 2015; Qin et al., 2016; Wu, 2017; Yang, 2017; Chang et al., 2018; Xu and Qi, 2018; Yang and Wang, 2018; Guan et al., 2019; Liu, 2019; Sun et al., 2019; Wen and Ding, 2019; Xu et al., 2019; Zhu and Li, 2019; Zuo, 2019; Zhang, 2020).

#### KPS

**FIGURE 4:**
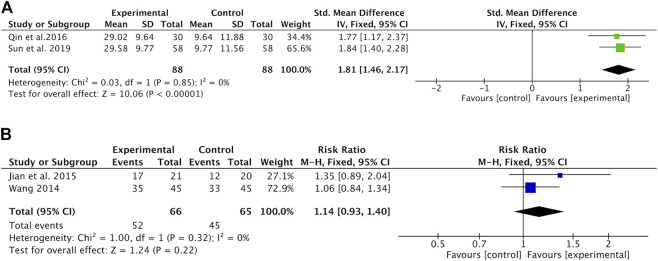
Forest plot of KPS of Astragalus-containing CHM combined with CT versus CT alone; outcomes: **(A)** mean ± SD of KPS; **(B)** the number of patients with nondeterioration KPS. KPS, Karnofsky performance score; CHM, Chinese herbal medicine; CT, chemotherapy.

The changes in KPS were reported as two types of data in the included studies: the mean ± SD of KPS before and after treatment and the number of patients who reported an improved or stable performance status based on KPS (ten-point cutoff). The value of KPS was recorded in two studies (Qin et al., 2016; Sun et al., 2019) with 176 patients. Meta-analysis showed that the KPS was significantly higher in the Astragalus-containing CHM combined with CT group than in the CT alone group (SMD = 1.81, 95% CI: 1.46–2.17, *p <* 0.00001) (Figure 4A). There was no significant heterogeneity among these studies (*I* ^*2*^ = 0%). Nondeterioration KPS was recorded in two studies (Wang, 2014; Jian et al., 2015) that included 131 patients. The results of the meta-analysis showed that there was no significant difference between the two groups (RR = 1.14, 95% CI: 0.93–1.40, *p* = 0.22) (Figure 4B). No significant heterogeneity existed among these studies (*I* ^*2*^ = 0%).

#### Reduction in CT Toxicity

**FIGURE 5:**
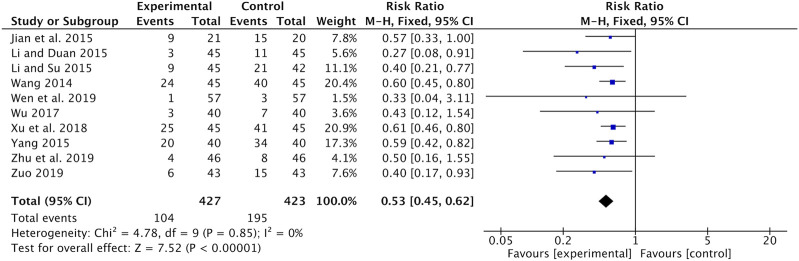
Forest plot of nausea and vomiting of Astragalus-containing CHM combined with CT vs. CT alone. CHM, Chinese herbal medicine; CT, chemotherapy.

Nausea and vomiting were recorded in ten studies (Wang, 2014; Jian et al., 2015; Li and Duan, 2015; Li and Su, 2015; Yang, 2015; Wu, 2017; Xu and Qi, 2018; Wen and Ding, 2019; Zhu and Li, 2019; Zuo, 2019). Meta-analysis showed that the incidence of nausea and vomiting was significantly lower in the Astragalus-containing CHM combined with CT group than in the CT alone group (RR = 0.53, 95% CI: 0.45–0.62, *p <* 0.00001, *I* ^*2*^ = 0%) (Figure 5).

**FIGURE 6:**
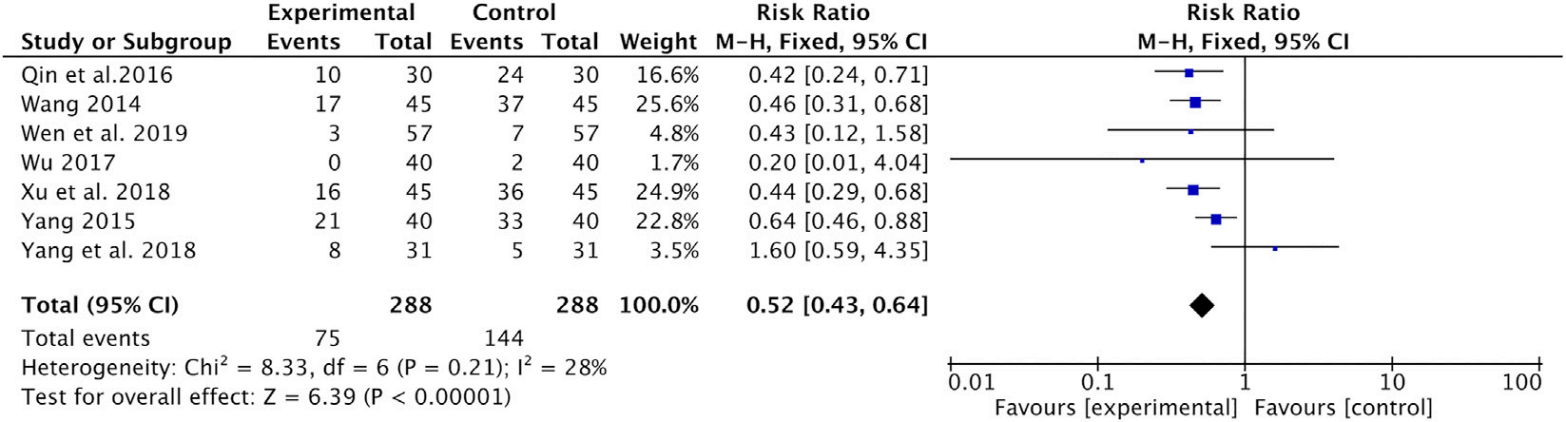
Forest plot of hair loss of Astragalus-containing CHM combined with CT versus CT alone. CHM, Chinese herbal medicine; CT, chemotherapy.

Hair loss was reported in seven studies (Wang, 2014; Yang, 2015; Qin et al., 2016; Wu, 2017; Xu and Qi, 2018; Yang and Wang, 2018; Wen and Ding, 2019). Meta-analysis indicated that the incidence of hair loss was significantly lower in the Astragalus-containing CHM combined with CT group than in the CT alone group (RR = 0.52, 95% CI: 0.43–0.64, *p* < 0.00001, *I* ^*2*^ = 28%) (Figure 6).

**FIGURE 7:**
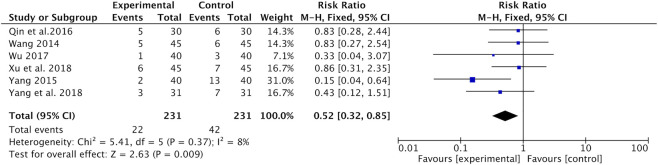
Forest plot of neurotoxicity of Astragalus-containing CHM combined with CT vs. CT alone. CHM, Chinese herbal medicine; CT, chemotherapy.

Neurotoxicity was recorded in six studies (Wang, 2014; Yang, 2015; Qin et al., 2016; Wu, 2017; Xu and Qi, 2018; Yang and Wang, 2018). Meta-analysis found that the incidence of neurotoxicity was significantly lower in the Astragalus-containing CHM combined with CT group than in the CT alone group (RR = 0.52, 95% CI: 0.32–0.85, *p* = 0.009, *I* ^*2*^ = 8%) (Figure 7).

**FIGURE 8:**
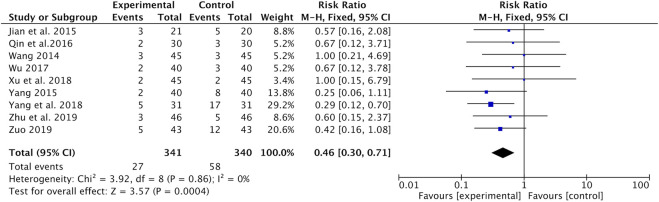
Forest plot of hepatic and renal toxicity of Astragalus-containing CHM combined with CT vs. CT alone. CHM, Chinese herbal medicine; CT, chemotherapy.

Hepatic and renal toxicities were recorded in nine studies (Wang, 2014; Jian et al., 2015; Yang, 2015; Qin et al., 2016; Wu, 2017; Xu and Qi, 2018; Yang and Wang, 2018; Zhu and Li, 2019; Zuo, 2019). Results of the meta-analysis reported that the incidence of hepatic and renal toxicity was significantly lower in the Astragalus-containing CHM combined with CT group than in the CT alone group (RR = 0.46, 95% CI: 0.30–0.71, *p* = 0.0004, *I* ^*2*^ = 0%) (Figure 8).

### Publication Bias

**FIGURE 9:**
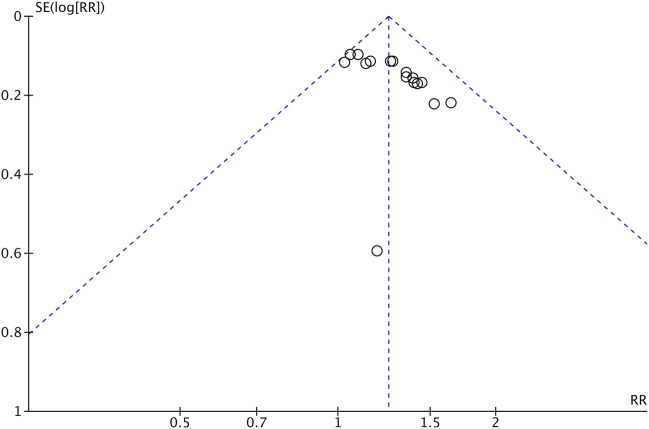
Funnel plot of tumor response (CR + PR) of Astragalus-containing CHM combined with CT vs. CT alone. CHM, Chinese herbal medicine; CT, chemotherapy; CR, complete response; PR, partial response.

We applied a series of strategies to investigate potential publication biases. Figure 9 presents a funnel plot of tumor response in the meta-analysis. The plot was symmetrical, which suggests that publication bias was not obvious.

## Discussion

Astragalus-containing CHM combined with CT is a popular complementary and alternative therapy used for cancer patients because it can increase therapeutic effects and decrease side effects (McCulloch et al., 2006). In recent years, some meta-analyses have been conducted to determine the clinical efficacy of Astragalus-containing CHM for non-small-cell lung cancer, breast cancer, colorectal cancer, and gastric cancer (Yao et al., 2014; Wang et al., 2016; Lin et al., 2019; Liu et al., 2019). Cervical cancer is a common prevalent disease in women. Despite advances in early screening methods, subtle symptoms can result in patients not going to a doctor until the cancer has progressed to an advanced stage, thereby delaying treatment. Astragalus-containing CHM has become the focus of several studies on cancer due to its low toxic side effects (Auyeung et al., 2016; Yang et al., 2020), but there is no systematic evidence of whether it is also applicable to cervical cancer. Therefore, we conducted a meta-analysis of the efficacy of Astragalus-containing CHM combined with CT for the treatment of cervical cancer. To our knowledge, this is the first systematic review and meta-analysis of RCTs on the efficacy of Astragalus-containing CHM in cervical cancer. This meta-analysis evaluated outcomes such as tumor response, quality of life (KPS), and chemotoxicity. A total of 19 studies were included in this review, involving 1,649 patients (827 in the Astragalus-containing CHM combined with CT group and 822 in the CT alone group).

The results of the meta-analysis show that tumor response significantly improves with Astragalus-containing CHM combined with CT. According to previous reports, the aqueous extract of *Astragalus membranaceus* has been shown to induce apoptosis of H22 tumor cells and inhibit tumor growth (Li et al., 2012). Polysaccharides from *Astragalus membranaceus* show potent immunomodulatory activity by stimulating macrophages and increasing the level of cytokines, including the tumor necrosis factor-alpha (TNF-α) and granulocyte-macrophage colony-stimulating factor (GM-CSF) (Zhao et al., 2011). Previous studies have demonstrated that treatment with Astragalus-containing CHM (SH003) reduces the levels of expression for G1 phase-related CDK (CDK2, CDK4, and CDK6) and cyclin D and induces extrinsic cell apoptosis in HeLa cervical cancer cells (Lee et al., 2017). Jin et al. (2014) reported that formononetin, a phytoestrogen from the root of *Astragalus membranaceus* and an *O*-methylated isoflavone, inhibits the growth of tumors from the human cervical cancer cell line HeLa, which mainly depends on Akt inactivation and caspase-3 activation. In addition, Duan and Wang (2002) conducted a clinical study on the effect of Astragalus in efficacy enhancing and toxicity reducing of CT in patients with malignant tumors; the results show that Astragalus could inhibit the tumor development, which is consistent with our study results.

CT has many side effects; therefore, it is necessary to find complementary and alternative approaches to help reduce them. In this meta-analysis, Astragalus-containing CHM combined with CT significantly reduced the side effects caused by CT. Astragalus-containing CHM usually comprises multiple herbs of natural origin. Phytochemicals and herbal mixtures act multispecifically by attacking multiple targets at the same time (Efferth et al., 2017). Multitarget therapies have been suggested to overcome the resistance of anticancer drugs. In particular, polypharmacology has been shown to have more advantages than drug combination for the reduction of side effects and selectivity for cancer cells (Xie and Bourne, 2015). We hypothesize that these features of Astragalus-containing CHM make it a good candidate for the treatment of cervical cancer.

The present study has some limitations. First, although we conducted a comprehensive search, all included RCTs were only carried out in China. For this reason, our results might not apply to populations in other parts of the world. Second, the methodological quality of the included RCTs was generally poor. Thirteen RCTs reported having used “random number tables,” while the remaining six RCTs only mentioned “randomization” without providing further details. Allocation concealment and blinding were not reported in any of the included RCTs. In addition, none of the included studies reported follow-up or drop-out rates, thereby displaying methodological flaws that might create biases. Consequently, our results should be interpreted with great caution. Third, no placebo was used in any of the included RCTs. The characteristics of Astragalus-containing CHM, such as a strong taste and smell, can create difficulties when making a placebo, especially in decoctions. Fourth, because most of the studies did not provide data on long-term follow-up, long-term efficacy was not evaluated. Lastly, none of the included studies reported approval of their experiments by responsible ethical committees. Considering the importance of protecting the rights of patients, complementary and alternative medicine researchers must develop an awareness of ethical issues.

In conclusion, the results of our systematic review and meta-analysis provide evidence of the efficacy of Astragalus-containing CHM in the treatment of cervical cancer. Astragalus-containing CHM might be a potential option to enhance the curative efficacy and reduce side effects of CT. However, because most of the included studies had low quality, the results should be interpreted with caution. To provide stronger evidence for the use of Astragalus-containing CHM in cervical cancer, high-quality rigorous RCTs will be needed in the future.

## Data Availability

The original contributions presented in the study are included in the article/Supplementary Material; further inquiries can be directed to the corresponding authors.
