# Supplementary material for: Astragalus-Containing Chinese Herbal Medicine Combined With Chemotherapy for Cervical Cancer: A Systematic Review and Meta-Analysis
Source: Front Pharmacol. 2021 Jul 30;12:587021. doi: 10.3389/fphar.2021.587021 (PMC8361476; doi:10.3389/fphar.2021.587021)
Supplement: Supplementary file 1 [file Table1.DOCX]

**Table S1**. Herbal ingredients used in Chinese herbal medicine formulas in the included studies.

| Author, Year | Chinese herbal medicine formulas | Herbal ingredients |
| --- | --- | --- |
| Chang et al.  2018 [11] | Gui Zhi Fu Ling decoction | Cinnamomum cassia Presl (Gui Zhi), Poria cocas (Fu Ling), Peach Kernel (Tao Ren), Paeonia veitchii (Chi Shao), Moutan Radicis Cortex (Mu Dan Pi), Curcuma Zedoary (E Zhu), Rhizoma Sparganii (San Leng), Pelodiscis Carapax (Bie Jia), Astragalus membranaceus (Huang Qi), Glycyrrhizae radix (Gan Cao). Based on syndrome differentiation. |
| Guan et al.  2019 [12] | Self-made Qing Gan Li Shi decoction | Glycyrrhizae radix (Gan Cao), Radix Bupleuri (Chai Hu), Astragalus membranaceus (Huang Qi), Codonopsis Pilosulae (Dang Shen), Pinellia ternata (Ban Xia), Dried ginger (Gan Jiang), Atractylodes macrocephala (Bai Zhu), Saposhnikovia divaricata (Fang Feng), Scutellaria baicalensis (Huang Qin), Evodia officinalis Dode (Wu Zhu Yu), Coptis chinensis (Huang Lian). |
| Jian et al.  2015 [13] | Modified Fu Zheng Xiao Zheng decoction | Astragalus membranaceus (Huang Qi), Codonopsis Pilosulae (Dang Shen), Atractylodes macrocephala (Bai Zhu), Ligustrum lucidum (Nu Zhen Zi), Lycii Fructus (Gou Qi Zi), Cuscuta semen (Tu Si Zi), Curcuma Zedoary (E Zhu), Coix Seed (Yi Yi Ren), Glabrous greenbrier rhizome (Tu Fu Ling), Wall lizard (Bi Hu), Paridis Rhizoma (Zhong Lou), Scutellaria barbata (Ban Zhi Lian), Cyathula officinalis Kuan (Chuan Niu Xi), Glycyrrhizae radix (Gan Cao). Based on syndrome differentiation. |
| Li and Duan 2015 [14] | Fu Zheng Pei Ben decoction | Coix Seed (Yi Yi Ren), Astragalus membranaceus (Huang Qi), Pseudostellaria heterophylla (Tai Zi Shen), Lycii Fructus (Gou Qi Zi), Ligustri Lucidi Fructus (Nü Zhen Zi), Salvia miltiorrhiza Bunge (Dan Shen), Cyathula officinalis Kuan (Chuan Niu Xi), Citri Reticulatae Pericarpium (Chen Pi), Atractylodes macrocephala (Bai Zhu). |
| Li and Su 2015 [15] | Fu Zheng Pei Ben decoction | Coix Seed (Yi Yi Ren), Astragalus membranaceus (Huang Qi), Pseudostellaria heterophylla (Tai Zi Shen), Lycii Fructus (Gou Qi Zi), Ligustri Lucidi Fructus (Nü Zhen Zi), Salvia miltiorrhiza Bunge (Dan Shen), Cyathula officinalis Kuan (Chuan Niu Xi), Citri Reticulatae Pericarpium (Chen Pi), Atractylodes macrocephala (Bai Zhu). |
| Liu 2019 [16] | Chinese herbal compound | Astragalus membranaceus (Huang Qi), Scutellaria barbata (Ban Zhi Lian), Cuscuta semen (Tu Si Zi), Codonopsis Pilosulae (Dang Shen), Curcuma Zedoary (E Zhu), Cyathula officinalis Kuan (Chuan Niu Xi), Ligustrum lucidum (Nu Zhen Zi), Glabrous greenbrier rhizome (Tu Fu Ling), Wall lizard (Bi Hu), Lycii Fructus (Gou Qi Zi), Glycyrrhizae radix (Gan Cao), Atractylodes macrocephala (Bai Zhu). |
| Qin et al. 2016 [17] | Fu Zheng Gu Ben decoction | Angelica sinensis (Dang Gui), Paeonia lactiflora (Bai Shao), American ginseng (Xi Yang Shen), Astragalus membranaceus (Huang Qi), Citri Reticulatae Pericarpium (Chen Pi), Pinellia ternata (Ban Xia), Fritillaria (Bei Mu), Glycyrrhizae radix (Gan Cao), Ziziphus jujuba (Da Zao). |
| Sun et al. 2019 [18] | Fu Zheng Gu Ben decoction | Angelica sinensis (Dang Gui), Paeonia lactiflora (Bai Shao), Astragalus membranaceus (Huang Qi), American ginseng (Xi Yang Shen), Fritillaria (Bei Mu), Pinellia ternata (Ban Xia), Glycyrrhizae radix (Gan Cao), Ziziphus jujuba (Da Zao). |
| Wang 2014 [19] | Fu Zheng Gu Ben decoction | American ginseng (Xi Yang Shen), Astragalus membranaceus (Huang Qi), Angelica sinensis (Dang Gui), Paeonia lactiflora (Bai Shao)，Citri Reticulatae Pericarpium (Chen Pi), Pinellia ternata (Ban Xia), Fritillaria (Bei Mu), Glycyrrhizae radix (Gan Cao). |
| Wen et al. 2019 [20] | Modified Ren Shen Yang Rong decoction | Ginseng radix (Ren Shen), Angelica sinensis (Dang Gui), Astragalus membranaceus (Huang Qi), Glycyrrhizae radix (Gan Cao), Atractylodes macrocephala (Bai Zhu), Poria cocas (Fu Ling), Paeonia lactiflora (Bai Shao), Polygala tenuifolia (Yuan Zhi), Rehmannia glutinosa (Shu Di), Schisandra chinensis (Wu Wei Zi), Citri Reticulatae Pericarpium (Chen Pi), Cinnamomi cassiae cortex (Rou Gui), Colla corii asini (E Jiao), Polygonatum sibiricum (Huang Jing)，Pheretima aspergillum (Di Long). |
| Wu 2017 [21] | Fu Zheng Gu Ben decoction | Glycyrrhizae radix (Gan Cao), Fritillaria (Bei Mu), Pinellia ternata (Ban Xia), Citri Reticulatae Pericarpium (Chen Pi), Paeonia lactiflora (Bai Shao), Angelica sinensis (Dang Gui), Astragalus membranaceus (Huang Qi), American ginseng (Xi Yang Shen). |
| Xu et al. 2018 [22] | Fu Zheng Gu Ben decoction | Glycyrrhizae radix (Gan Cao)，Astragalus membranaceus (Huang Qi), Paeonia lactiflora (Bai Shao), Angelica sinensis (Dang Gui), Citri Reticulatae Pericarpium (Chen Pi), American ginseng (Xi Yang Shen), Pinellia ternata (Ban Xia), Fritillaria (Bei Mu). |
| Xu et al. 2019 [23] | Fu Zheng Gu Ben decoction | Paeonia lactiflora (Bai Shao), Angelica sinensis (Dang Gui), Astragalus membranaceus (Huang Qi), American ginseng (Xi Yang Shen), Pinellia ternata (Ban Xia), Citri Reticulatae Pericarpium (Chen Pi), Fritillaria (Bei Mu), Ziziphus jujuba (Da Zao), Glycyrrhizae radix (Gan Cao). |
| Yang 2015 [24] | Fu Zheng Gu Ben decoction | Rehmannia glutinosa (Shu Di), Angelica sinensis (Dang Gui), Astragalus membranaceus (Huang Qi), Glycyrrhizae radix (Gan Cao), Roasted Rhizoma Atractylodes macrocephala (Chao Bai Zhu), Ginger (Sheng Jiang), Ziziphus jujuba (Da Zao), Ginseng radix (Ren Shen). |
| Yang 2017 [25] | Modified Ren Shen Yang Rong decoction | Astragalus membranaceus (Huang Qi), Ginseng radix (Ren Shen), Poria cocas (Fu Ling), Atractylodes macrocephala (Bai Zhu), Citri Reticulatae Pericarpium (Chen Pi), Paeonia lactiflora (Bai Shao), Angelica sinensis (Dang Gui), Rehmannia glutinosa (Shu Di), Polygonatum sibiricum (Huang Jing), Polygala tenuifolia (Yuan Zhi), Schisandra chinensis (Wu Wei Zi), Cinnamomi cassiae cortex (Rou Gui), Glycyrrhizae radix (Gan Cao). |
| Yang et al. 2018 [26] | San Xian Bao Gong decoction | Agrimonia pilosa (Xian He Cao), Astragalus membranaceus (Huang Qi), Oldenlandia diffusa (Bai Hua She She Cao), Crassostrea gigas (Mu Li), Epimedii Herba (Yin Yang Huo), Lycii Fructus (Gou Qi Zi), Curculigo orchioides Gaertner (Xian Mao Gen), American ginseng (Xi Yang Shen), Curcuma Zedoary (E Zhu), Fructus amomi (Sha Ren), Folium Perillae (Zi Su Ye), Notoginseng Radix et Rhizoma (San Qi), Hominis placenta (Zi He Che). |
| Zhu et al. 2019 [27] | Fu Zheng Yi Liu decoction | Astragalus membranaceus (Huang Qi), Scutellaria barbata (Ban Zhi Lian), Oldenlandia diffusa (Bai Hua She She Cao), Curcuma Zedoary (E Zhu), Codonopsis Pilosulae (Dang Shen), Atractylodes macrocephala (Bai Zhu), Poria cocas (Fu Ling), Citrus aurantium (Zhi Qiao), Magnolia officinalis (Hou Po), Armeniaca mume Sieb (Lü E Mei), Polygonum cuspidatum (Hu Zhang), Coix Seed (Yi Yi Ren), Fructus amomi (Sha Ren), Roasted foxtail millet (Chao Gu Ya). |
| Zuo 2019 [28] | Self-made Qing Gan Li Shi decoction | Astragalus membranaceus (Huang Qi), Pinellia ternata (Ban Xia), Glycyrrhizae radix (Gan Cao), Radix Bupleuri (Chai Hu), Codonopsis Pilosulae (Dang Shen), Dried ginger (Gan Jiang), Atractylodes macrocephala (Bai Zhu), Saposhnikovia divaricata (Fang Feng), Scutellaria baicalensis (Huang Qin), Evodia officinalis Dode (Wu Zhu Yu), Coptis chinensis (Huang Lian). |
| Zhang 2020 [29] | Gui Zhi Fu Ling decoction | Cinnamomum cassia Presl (Gui Zhi), Astragalus membranaceus (Huang Qi), Poria cocas (Fu Ling), Peach Kernel (Tao Ren), Moutan Radicis Cortex (Mu Dan Pi), Curcuma Zedoary (E Zhu), Rhizoma Sparganii (San Leng), Pelodiscis Carapax (Bie Jia), Glycyrrhizae radix (Gan Cao). |
