# Supplementary figures and images for: Astragalus-Containing Chinese Herbal Medicine Combined With Chemotherapy for Cervical Cancer: A Systematic Review and Meta-Analysis
Source: Front Pharmacol. 2021 Jul 30;12:587021. doi: 10.3389/fphar.2021.587021 (PMC8361476; doi:10.3389/fphar.2021.587021)

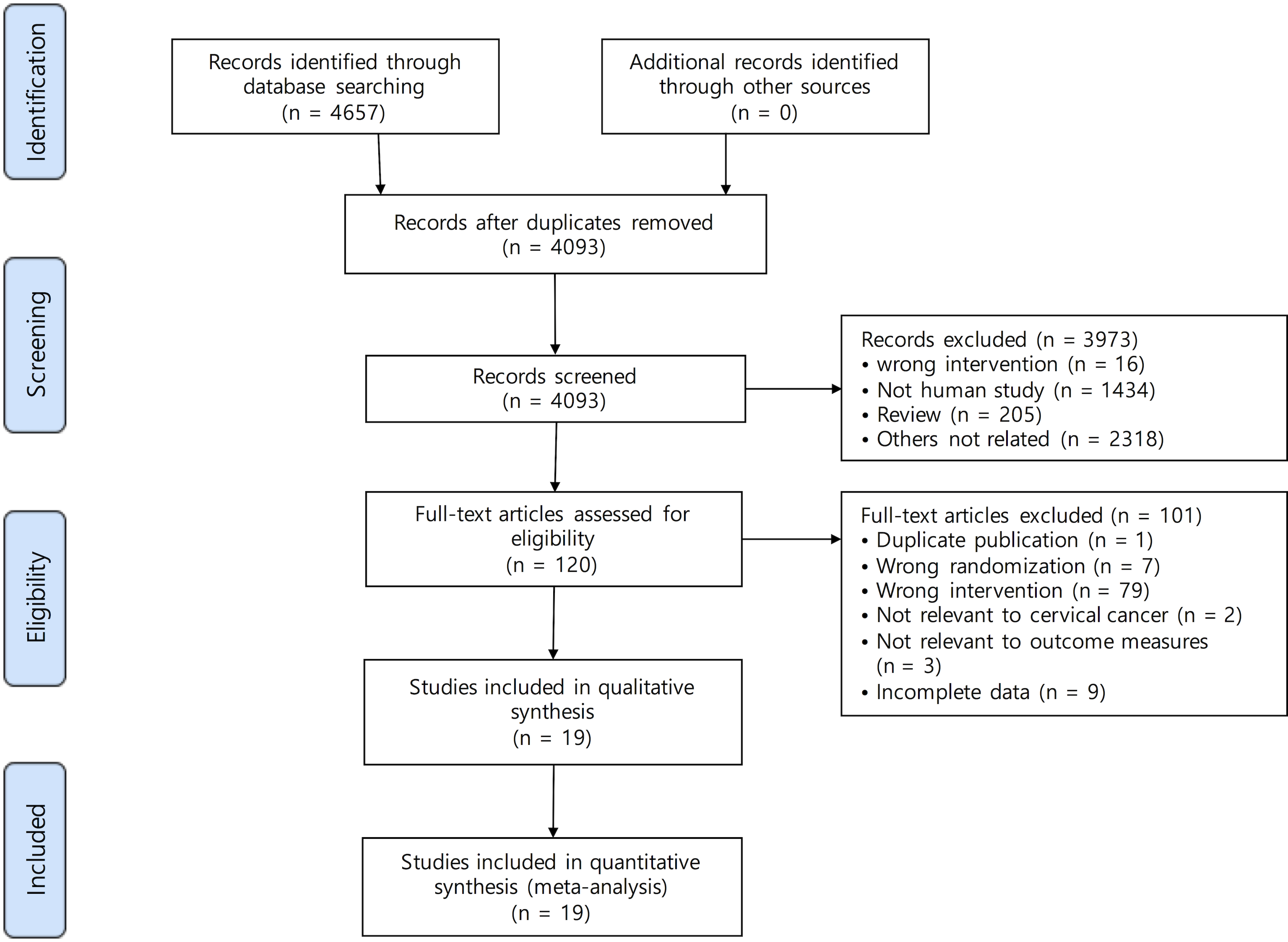

Supplement: Supplementary file 2 [file DataSheet1.ZIP › CC meta_Figure_200723/CC_meta_Figure 1_200723.tiff]

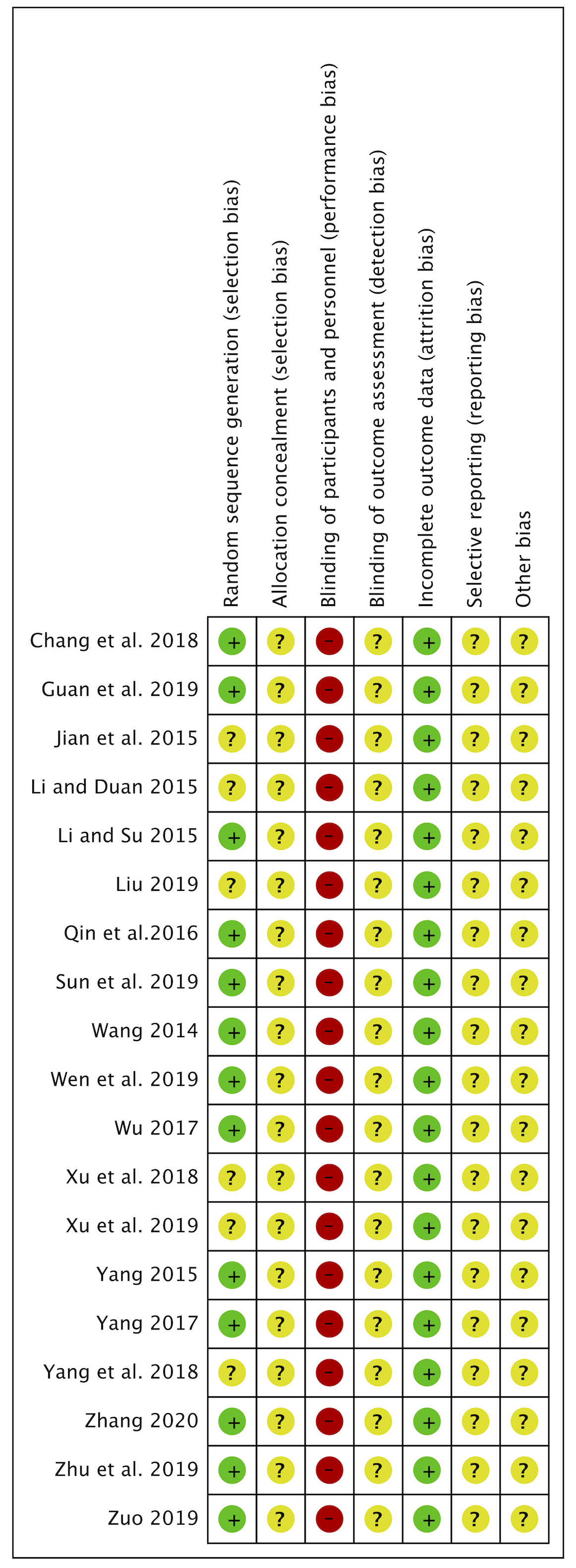

Supplement: Supplementary file 2 [file DataSheet1.ZIP › CC meta_Figure_200723/CC_meta_Figure 2_200723.tiff]

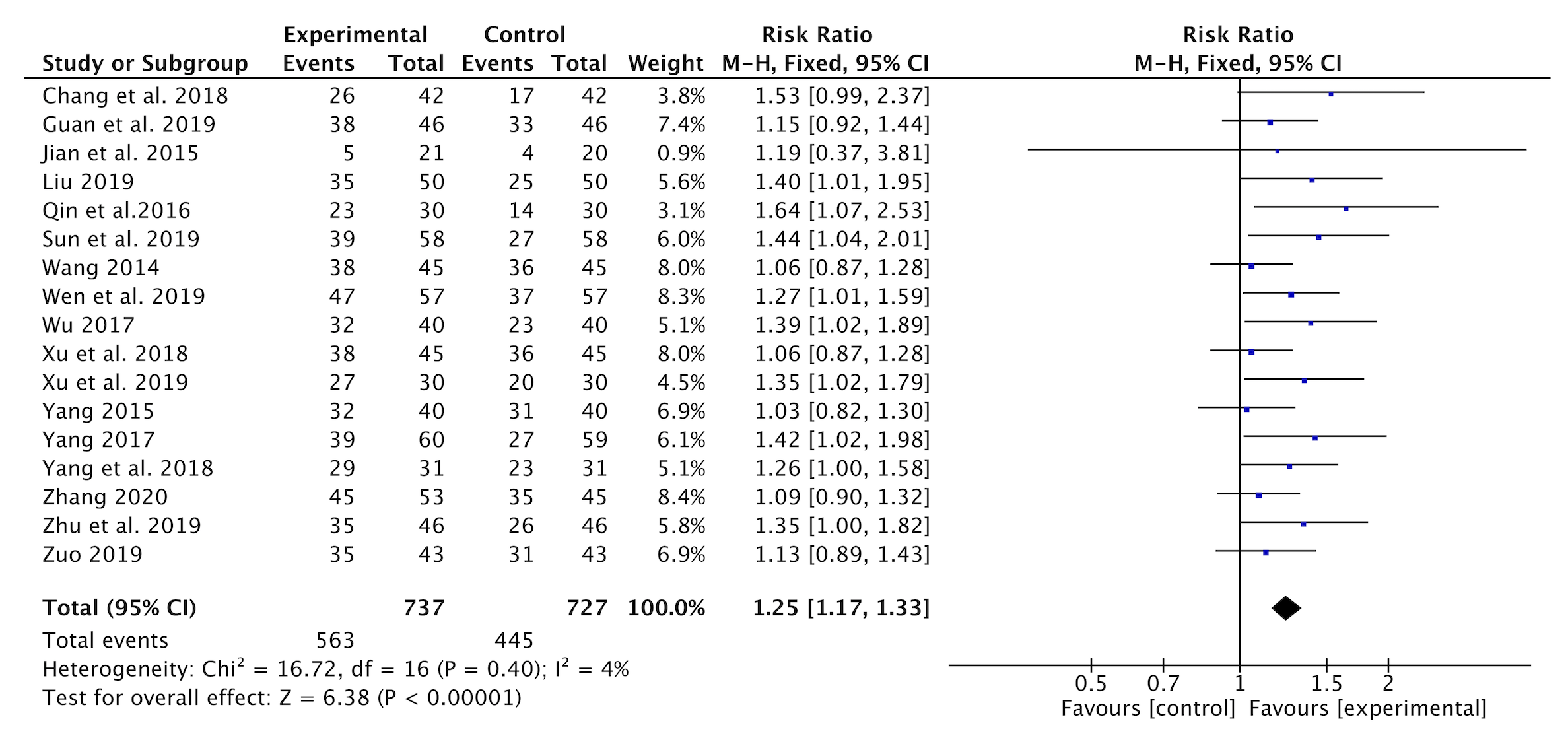

Supplement: Supplementary file 2 [file DataSheet1.ZIP › CC meta_Figure_200723/CC_meta_Figure 3_200723.tiff]

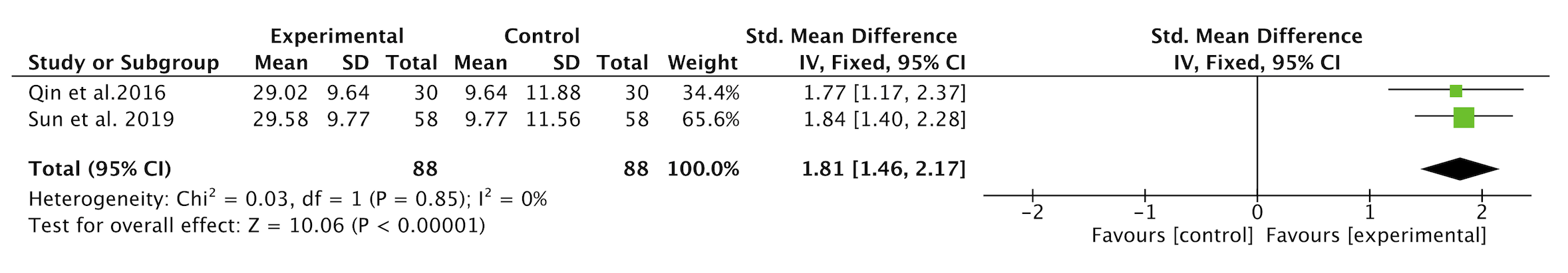

Supplement: Supplementary file 2 [file DataSheet1.ZIP › CC meta_Figure_200723/CC_meta_Figure 4A_200723.tiff]

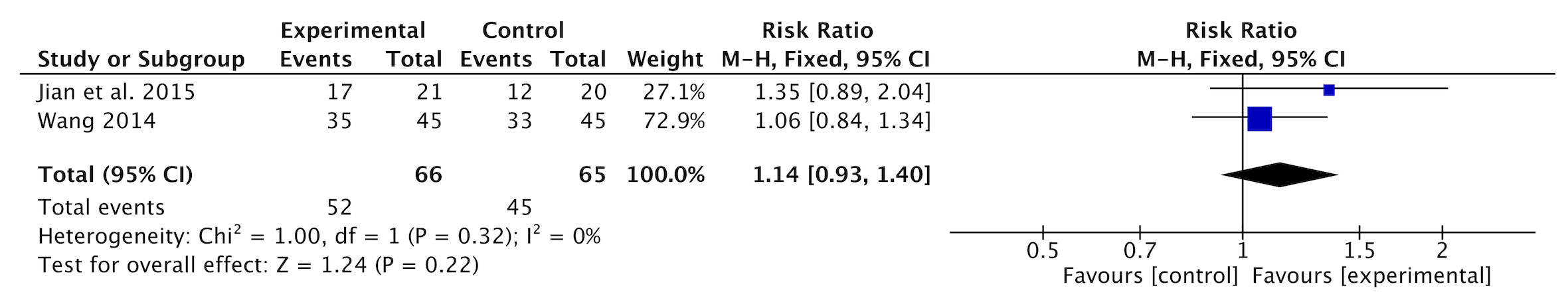

Supplement: Supplementary file 2 [file DataSheet1.ZIP › CC meta_Figure_200723/CC_meta_Figure 4B_200723.tiff]

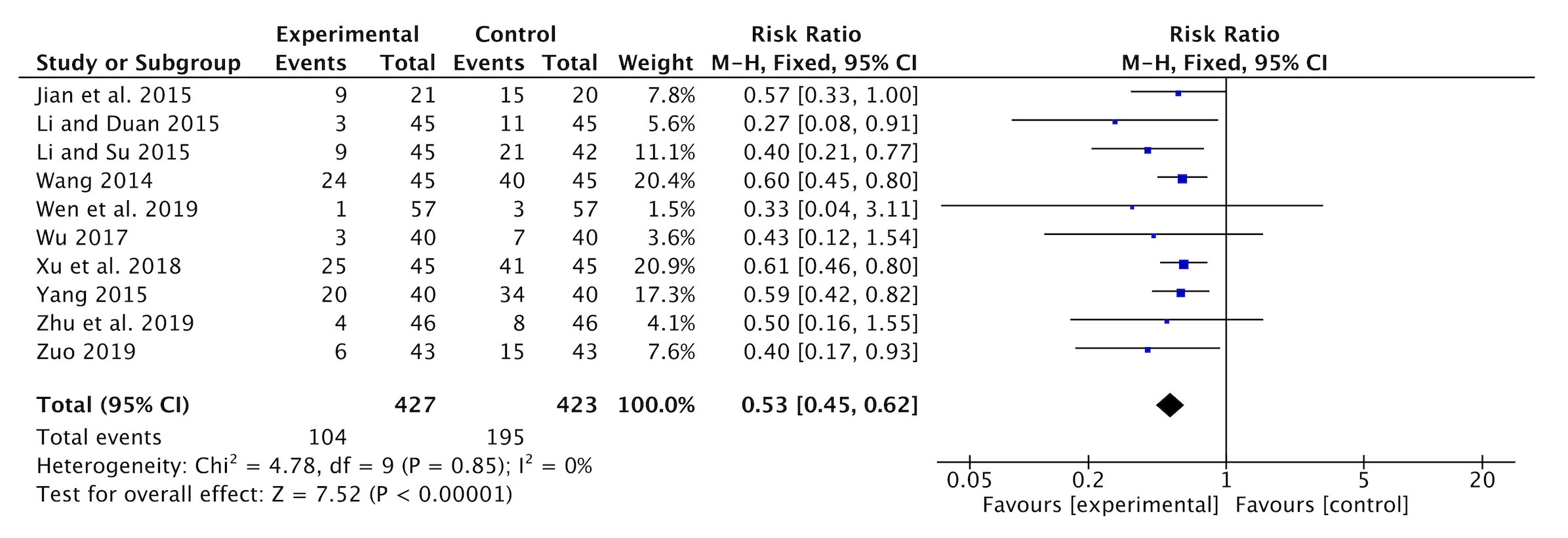

Supplement: Supplementary file 2 [file DataSheet1.ZIP › CC meta_Figure_200723/CC_meta_Figure 5_200723.tiff]

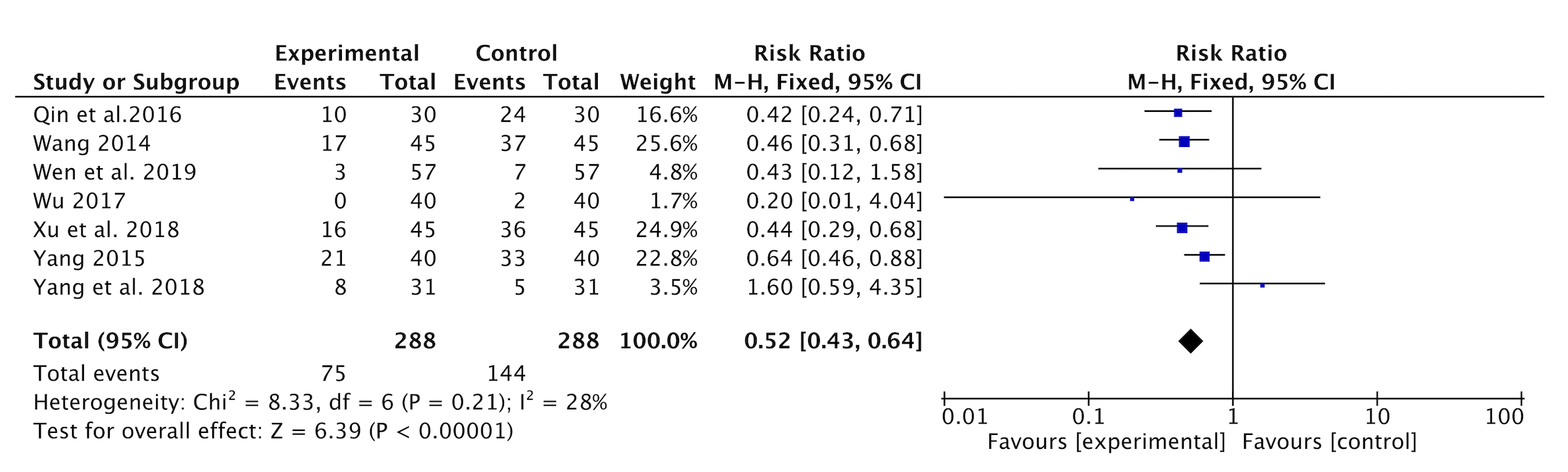

Supplement: Supplementary file 2 [file DataSheet1.ZIP › CC meta_Figure_200723/CC_meta_Figure 6_200723.tiff]

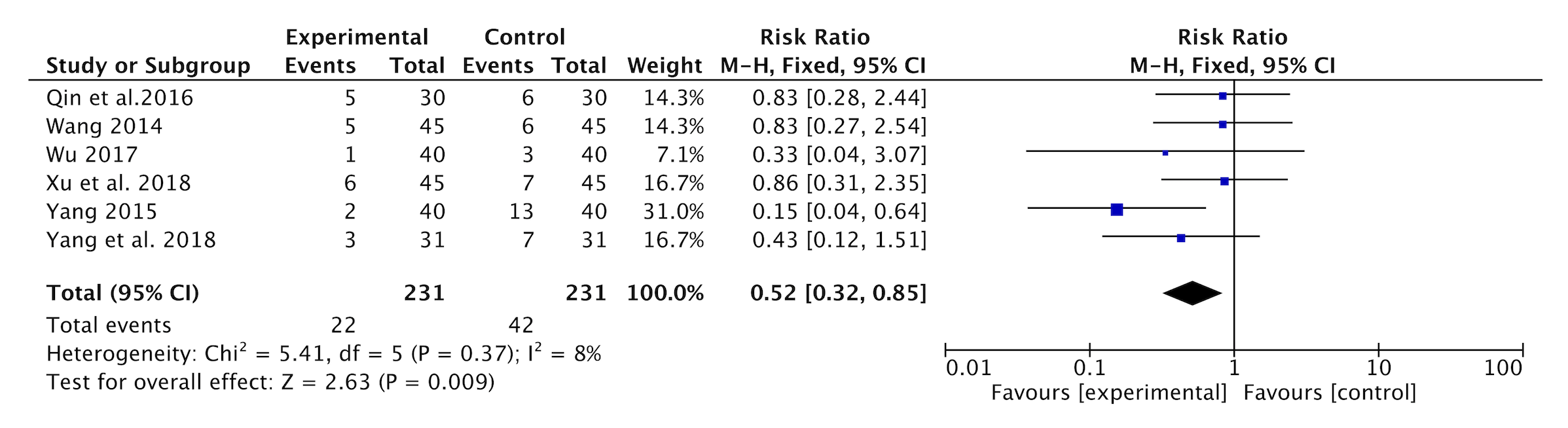

Supplement: Supplementary file 2 [file DataSheet1.ZIP › CC meta_Figure_200723/CC_meta_Figure 7_200723.tiff]

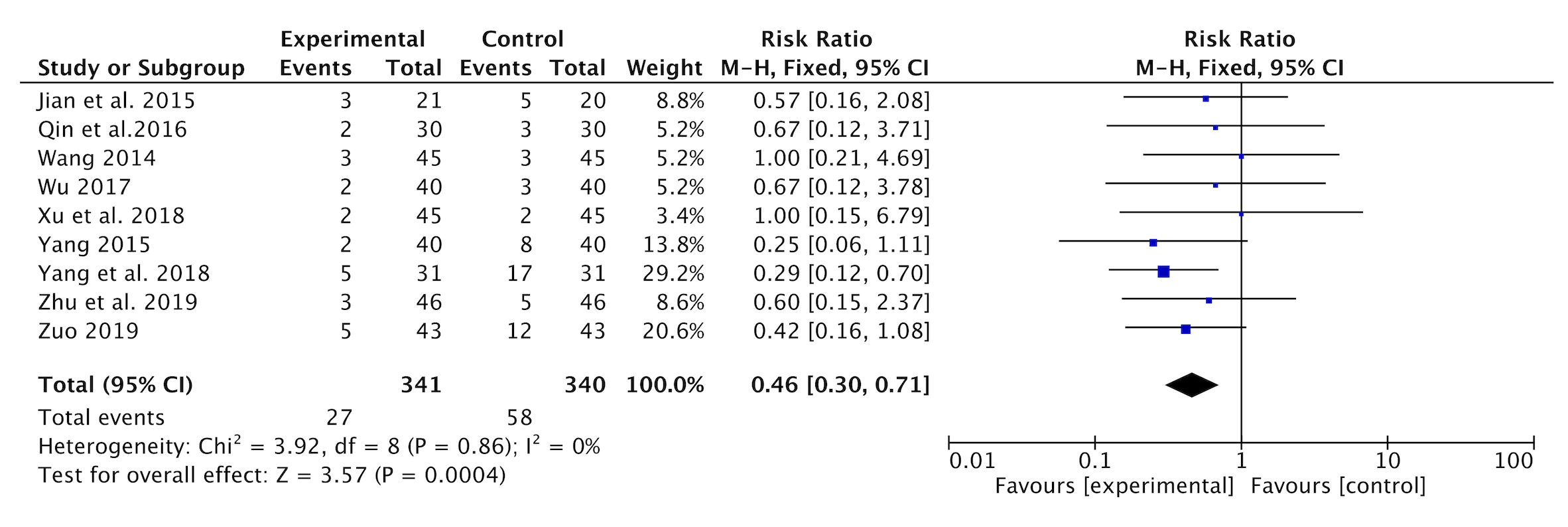

Supplement: Supplementary file 2 [file DataSheet1.ZIP › CC meta_Figure_200723/CC_meta_Figure 8_200723.tiff]

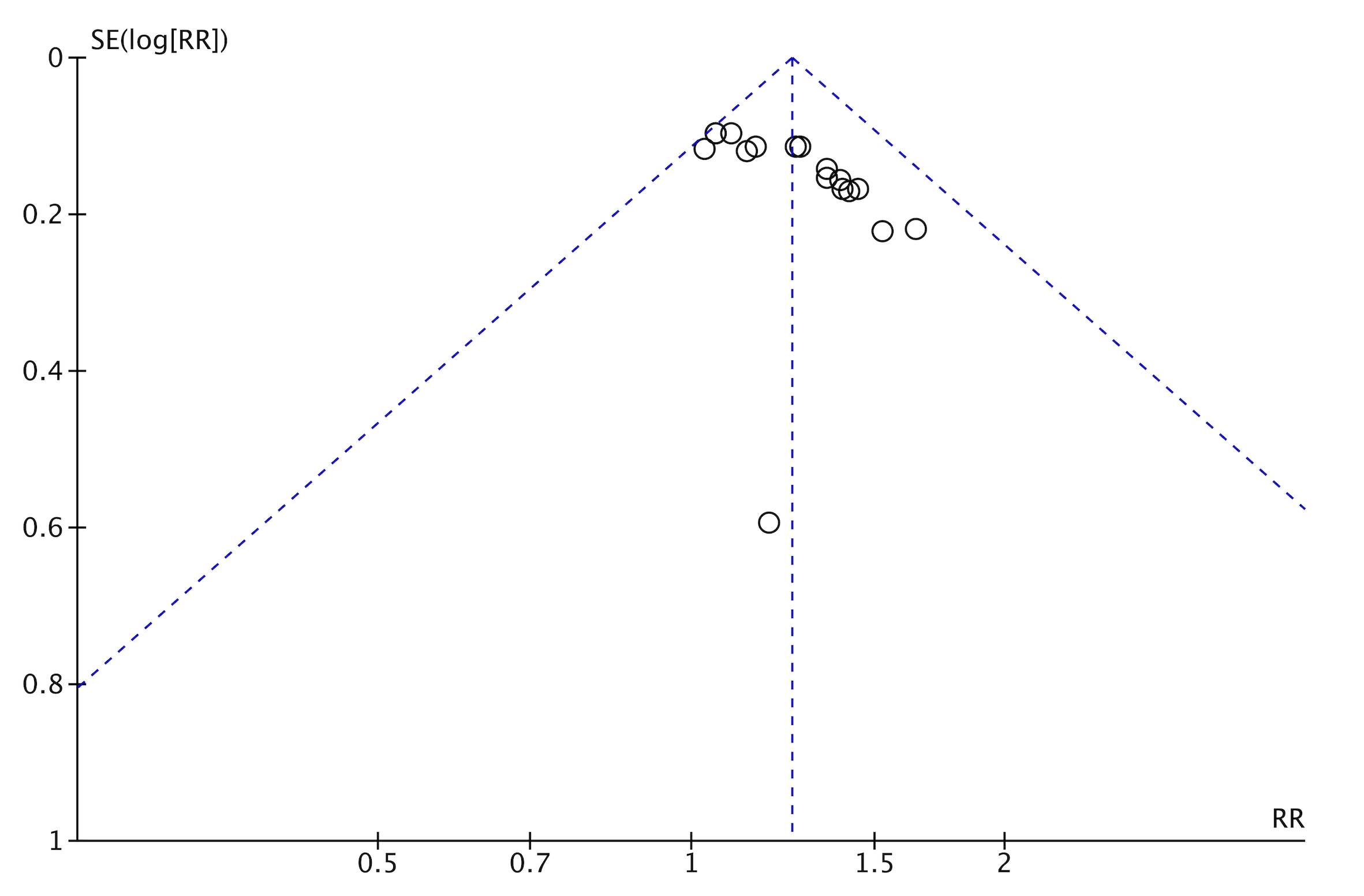

Supplement: Supplementary file 2 [file DataSheet1.ZIP › CC meta_Figure_200723/CC_meta_Figure 9_200723.tiff]
